# Supplementary material for: The interactome of intact mitochondria by cross-linking mass spectrometry provides evidence for coexisting respiratory supercomplexes
Source: Mol Cell Proteomics. 2017 Dec 8;17(2):216–32. doi: 10.1074/mcp.RA117.000470 (PMC5795388; doi:10.1074/mcp.RA117.000470)
Supplement: Supplemental Data [file supp_RA117.000470_133922_0_supp_23978_fzffwf.zip › spectra_annotation/mito_spectra_annotation/39-1-5-1-5-1.pdf]

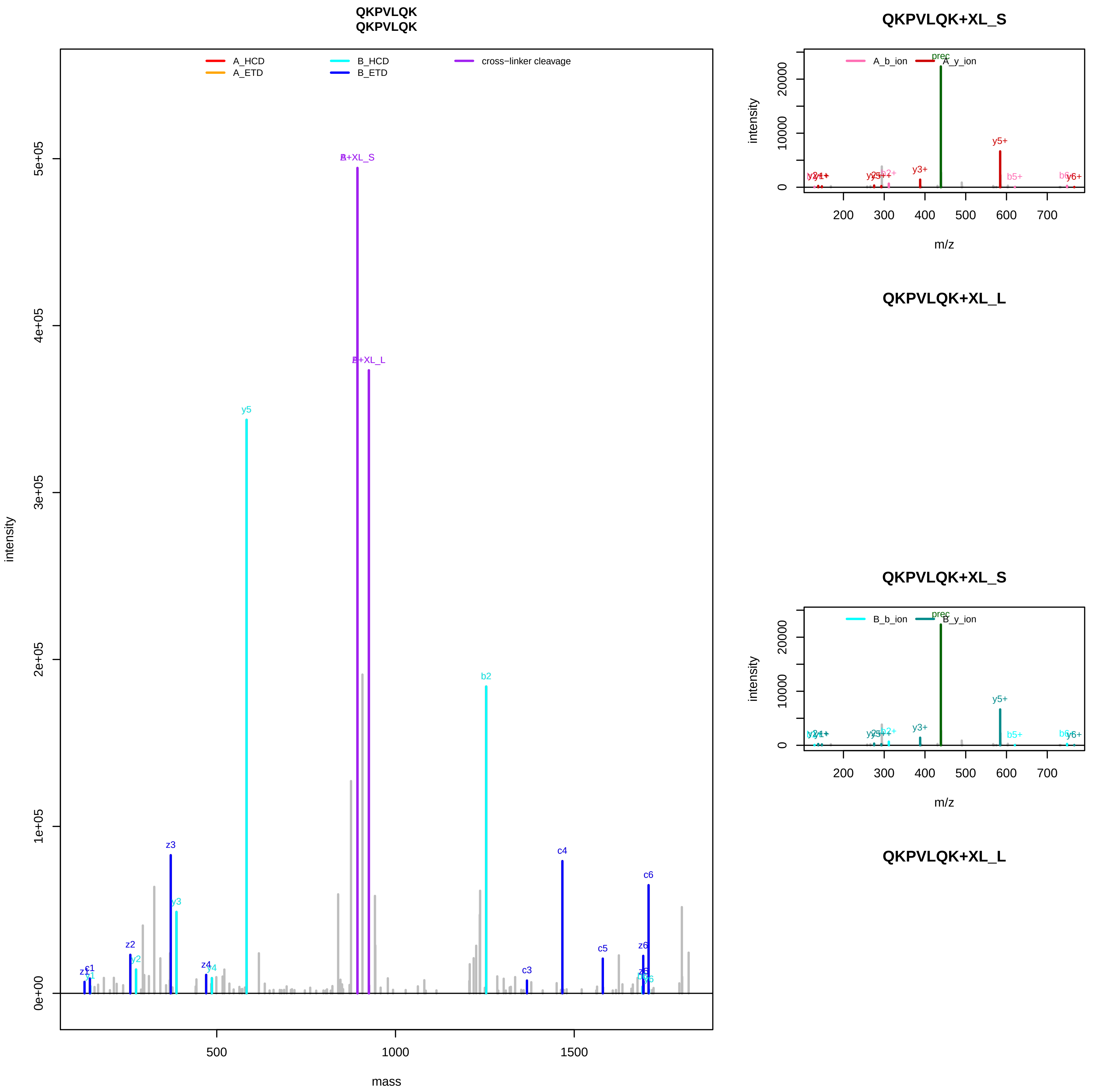
QKPVLQK
QKPVLQK
A_HCD
A_ETD
B_HCD
B_ETD
cross−linker cleavage
intensity
mass
A+XL_S
A+XL_L
y5
b2
z3
y3
c4
c6
c5
z6
z2
y2
z4
y4
c3
c1
z1
y1
0e+00
1e+05
2e+05
3e+05
4e+05
5e+05
500
1000
1500
QKPVLQK+XL_S
A_b_ion
A_y_ion
prec
y5+
y3+
b5+
y6+
m/z
200
300
400
500
600
700
0
10000
20000
QKPVLQK+XL_L
QKPVLQK+XL_S
B_b_ion
B_y_ion
prec
y5+
y3+
b5+
y6+
m/z
QKPVLQK+XL_L
